# Data describing the regional Industry 4.0 readiness index

**DOI:** 10.1016/j.dib.2020.106464

**Published:** 2020-10-27

**Authors:** Gergely Honti, Tímea Czvetkó, János Abonyi

**Affiliations:** a MTA-PE Complex Systems Monitoring Research Group, University of Pannonia, Veszprém, Hungary; b Institute of Advanced Studies Kőszeg, Chernel str. 14, H9730 Kőszeg, Hungary

**Keywords:** Industry 4.0, Indicator System, Open Data Analysis, News analysis, Regional Readiness, Regional Development, Digital Transformation, Innovation

## Abstract

The data article presents a dataset suitable to measure regional Industry 4.0 (I4.0+) readiness. The I4.0+ dataset includes 101 indicators with 248 958 observations, aggregated to NUTS 2 statistical level) based on open data in the field of education (ETER, Erasmus), science (USPTO, MA-Graph, GRID), government (Eurostat) and media coverage (GDELT). Indicators consider the I4.0-specific domain of higher education and lifelong learning, innovation, technological investment, labour market and technological readiness as indicators. A composite indicator, the I4.0+ index was constructed by the Promethee method, to identify regional rank regarding their I4.0 performance. The index is validated with economic (GDP) and innovation indexes (Regional Innovation Index).

## Specifications Table

**Table:** 

Subject	Management, Monitoring, Policy and Law
Specific subject area	Dataset for indicator-based monitoring of Industry 4.0 readiness
Type of data	Combined Data Table of the Industry 4.0 indicators, including on special joins (Data Table) Statistics of available I4.0 patents in the regions (Data Table) Statistics of available I4.0 publications in the regions (Data Table) Statistics of Erasmus+ program (Data Table) Statistics of Higher Education (Data Table) Statistics of Research Centers (Data Table) Statistics of Industry 4.0 relevant news from the media (Data Table) Rankings and indexes for validation (Data Table)
How data were acquired	Queried from Open Data portals, systematically joined and cleaned.
Data format	Raw data in CSV format
Parameters for data collection	Open Data sources were queried between 2008–2018, according to the aspects of Industry 4.0 regional development aspects, both pre- and post concept time-frame.
Description of data collection	Data is collected by systematic queries.
Data source location	ETER open data portal - Graduates in the fields of Industry 4.0 Erasmus+ open data portal - Mobility programs Microsoft Academic Graph open data portal - Publications GRID open data portal - Institutions USPTO open data portal – Patents EUROSTAT open data portal – Employment, population related data GDELT news analysis portal
Data accessibility	http://dx.doi.org/10.17632/23gwn43ygp.1

## Value of the Data

• The data is suitable to identify thematic areas as well as key indicators to measure the potential of the region in human capitals, the current development level high technology industries and manufacturing, investments and scientific outputs regarding regional Industry 4.0-related activity aggregated to city and the European Nomenclature of Territorial Units for Statistics level 2 (NUTS2) level.
• The developed composite indicator can function as a regional Industry 4.0 performance monitoring tool for decision makers and regional development researchers.
• The well curated, carefully mined and selected data is compiled and ready to analyse in multiple statistical software and in regional statistical software, to improve the thinking about the Industry 4.0 concept as well as to monitor the convergence towards it by merging the key aspects e.g. technology, investment, higher education into a uniformed reasonable as well as analyzable dataset.

## 1 Data Description

The collected dataset aims to identify the regional potential of Industry 4.0, covering five dimensions of Industry 4.0 regional development aspects, namely the Labour market, Technological readiness, Innovation, Investment and Higher education.

The data collection occurred through seven open data portals:

• European Tertiary Education Register (ETER) [1] – Higher education graduates, in Industry 4.0 relevant fields.
• Erasmus+ – Statistics about students participating in mobility programs [2].
• Microsoft Academic Knowledge Graph (MA-Graph) [3] – Publications about and in topic of Industry 4.0.
• Global Research Identifier Database (GRID) [4] – Worlds research organizations.
• United States Patent and Trademark Office (USPTO) [5] – American Patent database containing American and international patents.
• European Statistical Office (EUROSTAT) [6] – Containing population and economic data for the European regions.
• GDELT – Global News Monitoring Project offers 15 minutes dumps from 2015 until today, from the global media extracted [7].

The following databases provide a description of data included in the I4.0 index. The databases are joined together in the region level (NUTS2)

- selected_topics_i40_papers.csv: We have selected Industry 4.0 categorizations of scientific papers form Microsoft Academic Graphs ontology.
  ○ id (*string*) – Shows the identifier of the ontological element in the Microsoft Academic Knowledge Graph.
  ○ name (*string*) – The name of the topic.
  ○ include_subtopics (*bool*) – Indicator of including subgroups.
- selected_topics_i40_patents.csv: The selection of the Industry 4.0-relevant patent topics was done along the European standard of patent categorization.
  ○ id (*string*) – Shows the identifier of the patent category. This field corresponds to the CPC standard.
  ○ name (*string*) – Human readable name of the topic.
  ○ include_subtopics (*bool*) – Shows if the topic should include all subtopics or not.
- I40_indicator_db_column_description.csv: The database contains all the 101 indicators. Regarding its volume, we are sharing its description separately, in the description file, which includes the following:
  ○ ColumnName (*string*) – Describes the column name how to refer to the data.
  ○ DataType (*string*) – Contains the type of data in the column.
  ○ Description (*string*) – Description of the data.
- I40_indenticator_db.csv: Joined table of the previously mentioned data (I40_indicatior_db_column_description.csv).
- rankings.csv: This table describes the results of the different regional rankings.
  ○ Regio (*string*) – The region with NUTS2 encoding.
  ○ GDPrank (*int*) – Rank of the region based on GDP.
  ○ PrometheeRank (*int*) – The rank of the region by the promethee method.
  ○ RII (*float*) - Regional Innovation Index from 2019, by the Regional Innovation Scoreboard.

**Fig. 1:**
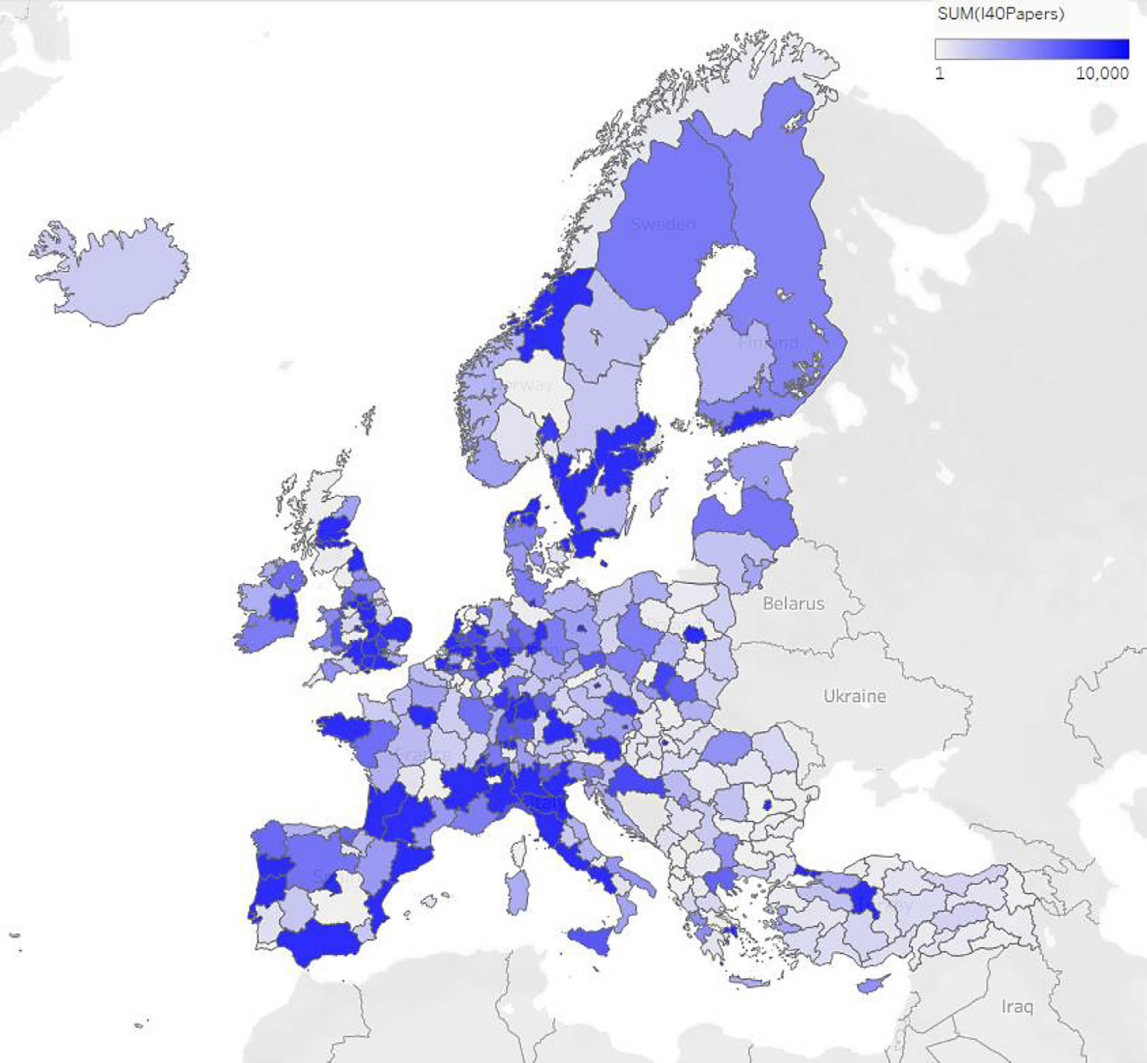
Scientific publications in the topic of Industry 4.0 by regions. The bluer the region is, the more publications are occurred in the topic.

**Fig. 2:**
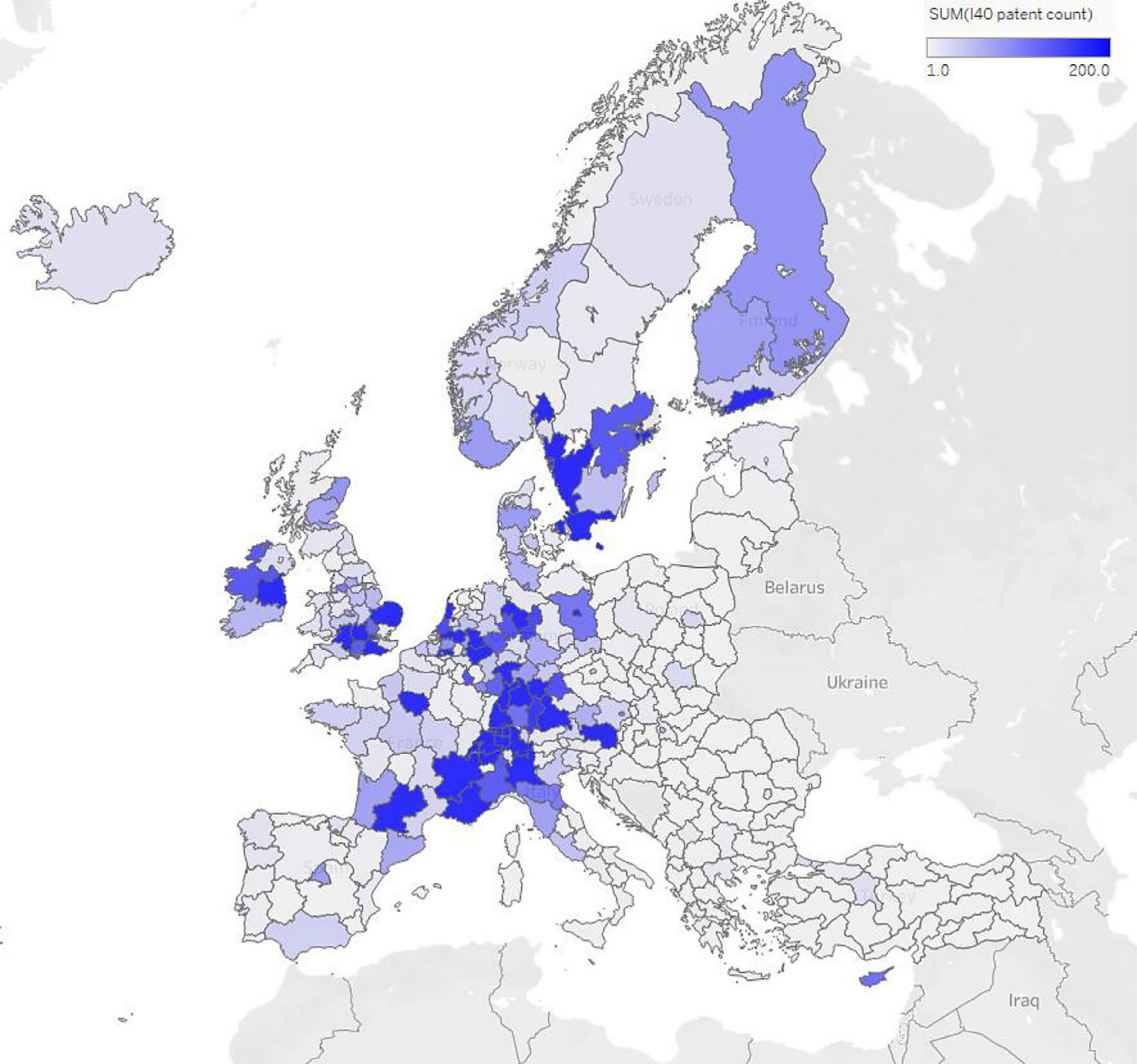
Patents in Industry 4.0 topics, by EU regions. The bluer a region is, the more patents it owns in the topic.

Governmental policies are operated in the long-term planning horizon by reflecting socio-economic as well as environmental development focused visions. It is stated that governmental policies focusing on application of Industry 4.0 simultaneously develop the region itself [8]. Macroeconomic open data proved to be suitable to measure regional innovation dynamics [9], inclusive growth [10] as well as socio-economic performance [11], however the I4.0-specific assessment has not been studied extensively. The collected, cleaned and analyzed dataset represents the socio-economical and technical standing of the current regional Industry 4.0 readiness. The collected data measures the potential and competitiveness of the region by the most crucial vectors, human capitals, as well as the current development level of high technology industries and manufacturing [12].

**Fig. 3:**
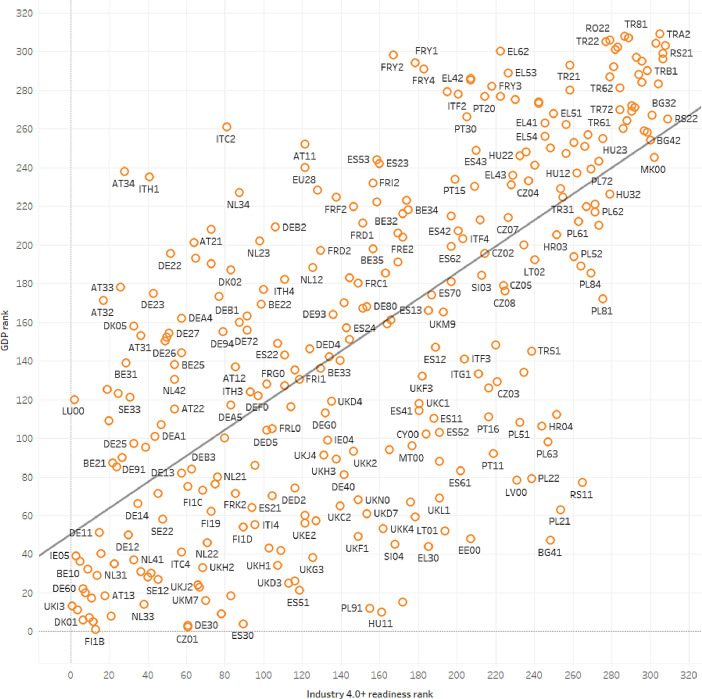
Correlation between GDP based regional rank and the Industry 4.0+ readiness rank $\left(r^{2}=0.4589\right)$.

**Fig. 4:**
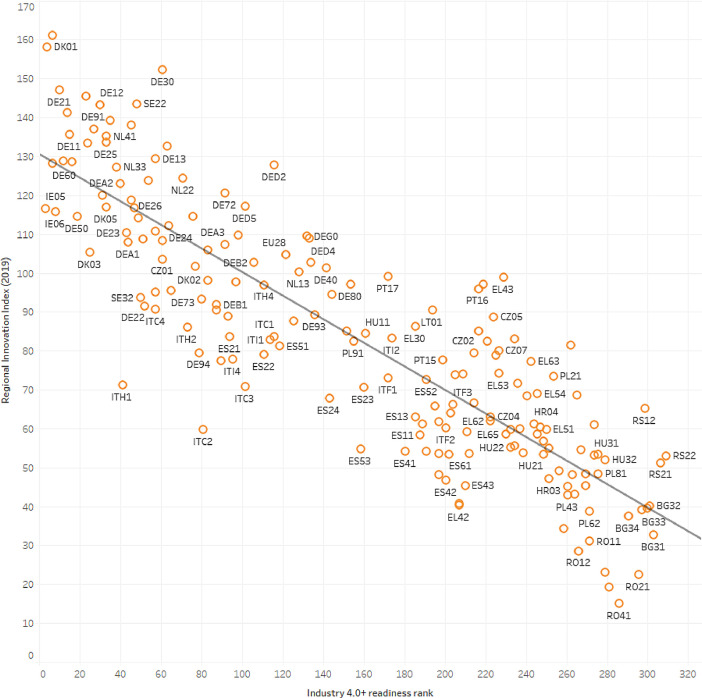
Correlation between the Regional Innovation Scoreboard Regional Innovation Index in year 2019, and the Industry 4.0+ readiness rank $\left(r^{2}=0.7502\right)$.

**Fig. 5:**
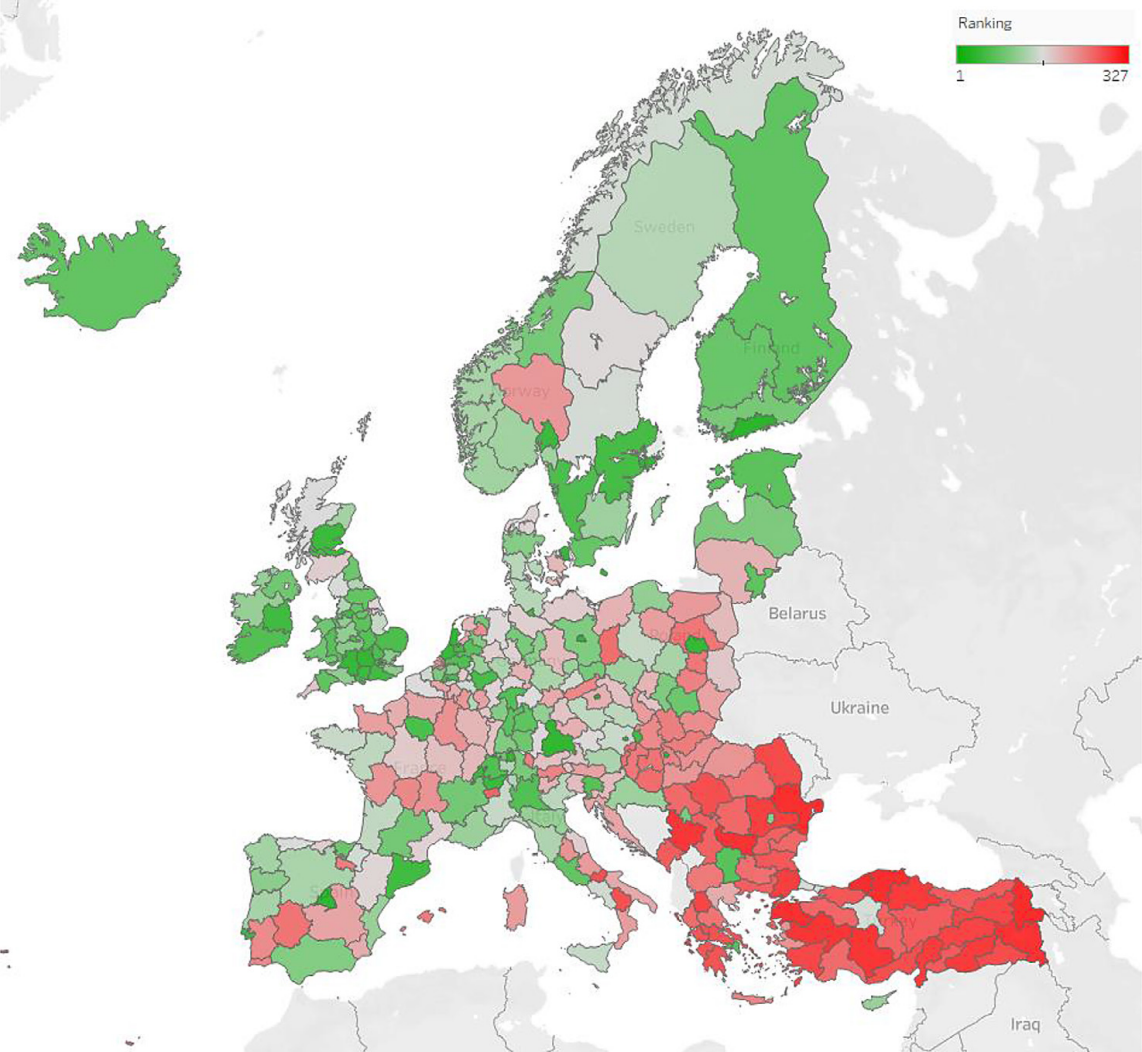
European Industry 4.0+ readiness rank leaderboard, based on indicators; The greener a region, the more it is ready for the adaption of Industry 4.0.

**Fig. 6:**
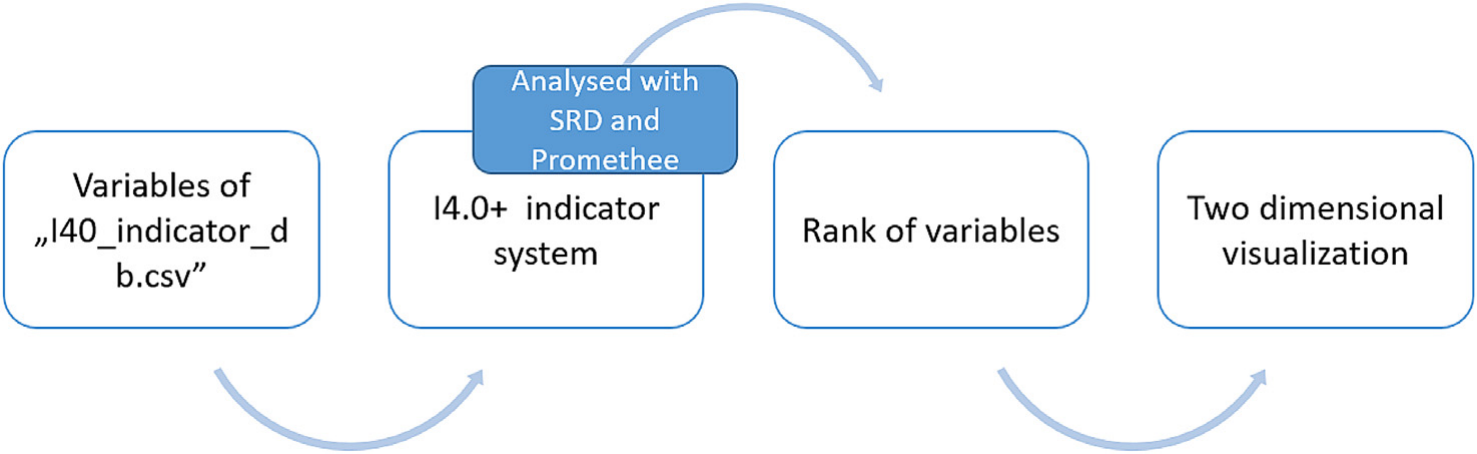
Analysing methodologies to determine correlations and the rank of variables.

The dataset reflects upon key components of the new industrial revolution, such as the occupation possibilities for every level of education, as well as the current industrial and scientific outputs as well as its investments. The dataset is also taking soft indicators into account, such as the opinions of the media on selected keywords strongly correlated with the new industrial revolution (e.g. “JOBS”, “MANUFACTURING”), where we measured the number of news appeared as well as the average sentiments of the texts. An informative, ranking system based on the collected indicators is provided, that can be effectively interpreted.

Fig. 1. shows the density of Industry 4.0-specific scientific publications in European regions. We notice that the locations of the major universities, as well as the highly advanced industrial regions, e.g. North Italy are the key contributors.

Fig. 2. shows the patent distribution in the relevant field across Europe. The previous scientific contributions are in this case not so significant anymore but the industrial competent of the region. We see that the main contributors are the advanced northern Italy and the Bayern region, known in the car industry as well as the southern part of Sweden.

We created an Index readiness 4.0+ rank, from the collected indicators and indexes, using the Promethee method [13]. Next, we show the correlations of the new rank with existing rankings and indexes.

Fig. 3. shows the correlation between GDP ranking and Industry 4.0+ readiness rank. The correlation is not so high as several factors and industries influence the GDP. However, this result illustrates that regions paying attention on science and technological employment has high GDP.

Fig. 4. indicates a 0.75 correlation between the I4.0+ and the Regional Innovation indexes. Their similarity is clear, however, the I4.0+ index measures only I4.0-specific areas that can boost regional innovation performance.

Fig. 5. shows the regions of Europe, colored according to their rank in the Industry 4.0+ readiness. According to the results, the most developed region is located in southern Finland, in Helsinki-Uusimaa region, followed by the region of the capital city of the Czech Republic, Prague and of Germany, Berlin.

## 2 Experimental Design and Methods

The problem of missing focus on regional Industry 4.0 readiness is studied through examining existing Industry 4.0 readiness models and indexes as well as exploring open data that is available at regional scale. To sufficiently measure regional Industry 4.0 (I4.0+) readiness, we defined the requirements of data to be: NUTS 2 classified (greater coverage of data), available (online), Industry 4.0-specific (direct metrics) and up-to-date. Therefore, data sources meets the criteria are identified as following: European Tertiary Education Register (ETER), Erasmus+, Microsoft Academic Knowledge Graph (MA-Graph), Global Research Identifier Database (GRID), United States Patents and Trademark Office (USPTO), European Statistical Office (Eurostat) and the Global Database of Events, Language and Tone (GDELT). News can serve as an effective tool for online monitoring without significant delay, for which GDELT provides a platform to extract and monitor world news by using natural-language and data-mining algorithms. It consists of the Event Database and the Global Knowledge Graph (GKG). The former captures events, while the latter records and connects locations, organizations, themes, people, taxonomies, sources, tone and event of news.

The indicators of ‘I40_indenticator_db.csv’ are categorized into five main dimensions, namely: higher education and lifelong learning, labour market, innovation, investment and technology readiness. Fig. 6. presents the methodological workflow of analysis. Variables are used to form the regional Industry 4.0 (I4.0+) indicator system, which was analysed with both SRD [14] and the Promethee II. [13] method. The result promoted the rank of variables, which is interpreted by the use of PCA method in a two dimensional visualization.

## CRediT authorship contribution statement

**Gergely Honti:** Data curation, Visualization, Visualization, Software, Software, Validation, Writing - original draft. **Tímea Czvetkó:** Conceptualization, Investigation, Visualization, Writing - original draft. **János Abonyi:** Conceptualization, Methodology, Software, Validation, Writing - original draft, Supervision.

## Declaration of Competing Interest

The authors declare that they have no known competing financial interests or personal relationships which have, or could be perceived to have, influenced the work reported in this article.
